# Doctors and Drunkards

**Published:** 1874-02

**Authors:** C. N. Hewitt


*THE N. W. MEDICAL AND SURGICAL JOURNAL—January,* 1874:

Doctors and Drunkards—*By C. N. Hewitt, M.D.*—It is proven:

First, That the propensity to drink may be transmitted from parent to offspring; that the drunkard may be the victim of a parent's sin, *i. e.* an hereditary drinker.

Second, That other diseased conditions of the body may create the appetite for stimulants.

Third, That even the temperate use of them may in peculiar constitutions develop the appetite into a controlling passion; and finally that these dispositions to drink occur in persons of both sexes and of every age and condition of life. They explain in the only practicable way why the rigid and prompt enforcement of legislation on the subject has so often failed hitherto in attaining its objects, which are, 1st, The maintenance of public order, 2d, The care and cure of the victims of stimulants.

It is to the credit of American medicine that American physicians were the first carefully to study this whole subject from the standpoint of science, and to originate methods of treatment which an experience of nearly twenty years has served to confirm and strengthen. Since the organization of the first inebriate asylum in America more than six thousand inebriates, most of them given up as lost, have been treated on the new plan, and of that number more than ninety-four per cent. were voluntary patients. Of the 6,000 more than 1,800, or thirty per cent., have been permanently cured; twelve per cent. decidedly benefited, and the remainder have declined further treatment, or have been given up as hopeless of cure. This great experience has enabled these experts in inebriety to establish the truth of the following propositions:

First, That the appetite for stimulants is a constitutional peculiarity of the inebriate, and that it may be inherited or acquired.

Second, That inebriety is a disease the result of that appetite, and is as amenable to treatment as any other disease.

Third, That it consists, essentially, in the loss of self-control or the power of the will, and in the substitution therefor of a blind impulse to the use of intoxicants, with the organic changes which these agents induce in the physical organization.

Fourth, That this morbid appetite results, unchecked, in the production, among some of its victims, of a veritable frenzy, "mania a potu," or of a monomania of suspicion, under the operation of either of which insane impulses, the victims may be guilty of the most atrocious crimes.

Fifth, That inebriates are of three great classes.

(a) Hereditary drunkards.

(b) Drunkards from causes not hereditary.

(c) Confirmed inebriates, which includes the hopeless cases of the other classes.

In all of these classes the appetite may manifest itself by habitual or periodic outbreaks.

Sixth. The periodic drinker is the most difficult to treat and most likely to remain cured. Cure is most likely between the ages of 35 and 45 years. The most hopeless cases are among the young, in whom the disease is, as a rule, hereditary. Cure is more likely among men than women, and among people of education and refinement than the reverse.

In this condensed statement is the result of years of patient trial and study.

The only rational and hopeful method with inebriates must begin with the recognition that *inebriety is a disease, not a crime,* and that the object of treatment must be not only the removal of the object of the appetite, the intoxicant, but the restoration of the self-respect and self-control of the patient.

This is to be done in institutions known as Homes, Retreats, or Asylums, where every means can be brought to bear in the most efficient way, for care and cure. It is not safe to trust to the voluntary action of the inebriate, but as a rule, he should be committed by due process of law to the institution, with as little publicity and shock to his feelings, as is consistent with due regard to his rights and liberty.

Though inebriety may result in hopeless insanity, it is not to be confounded with or treated as insanity. The mental difficulty is rather one of the will than of the understanding, and therefore requires different methods.

